# Zygomaticomaxillary Complex Fracture

**Published:** 2014-08-21

**Authors:** Srinivas M. Susarla, Zachary S. Peacock

**Affiliations:** ^a^Department of Plastic and Reconstructive Surgery, Johns Hopkins Hospital, Baltimore, Md; ^b^Department of Oral and Maxillofacial Surgery, Massachusetts General Hospital, Boston, Mass

**Keywords:** malar fracture, maxillofacial trauma, midface fracture, zygomaticomaxillary complex, orbitozygomatic fracture

## DESCRIPTION

A 43-year-old woman sustained left-sided facial injuries secondary to a mechanical fall from standing, while intoxicated. On evaluation, she complained of left-sided facial swelling, cheek numbness, and flattening of her cheek prominence.

## QUESTIONS

1. **What are the signs and symptoms associated with a zygomaticomaxillary complex (ZMC) fracture?**
2. **What are the indications for operative management for ZMC fractures?**
3. **What incisions are used to approach the ZMC?**
4. **When operatively managing a ZMC fracture, when is internal orbital reconstruction necessary?**

## DISCUSSION

Patients with ZMC fractures often present with tenderness, ecchymosis and edema over the malar prominence, lateral orbit, upper and lower eyelids, loss of malar projection, and blunting of the lateral canthus relative to the unaffected side.^1^ Medially displaced zygomatic arch fractures may impinge on the coronoid process or temporalis tendon, resulting in trismus. Paresthesia in the region of the infraorbital nerve is a common neurosensory finding. Significant involvement of the orbital floor may result in extraocular muscle dysfunction/entrapment, diplopia, or enophthalmos.^2^

Nondisplaced fractures can be managed expectantly.^1^ Patients should be advised to avoid activities that may result in facial injury and should remain on sinus precautions, a soft diet, and decongestants. While there is no strong evidence for the routine use of antibiotics for closed fractures, many surgeons will prescribe antibiotics for prophylactic sinus coverage for 5 to 7 days.

**Figure 1:**
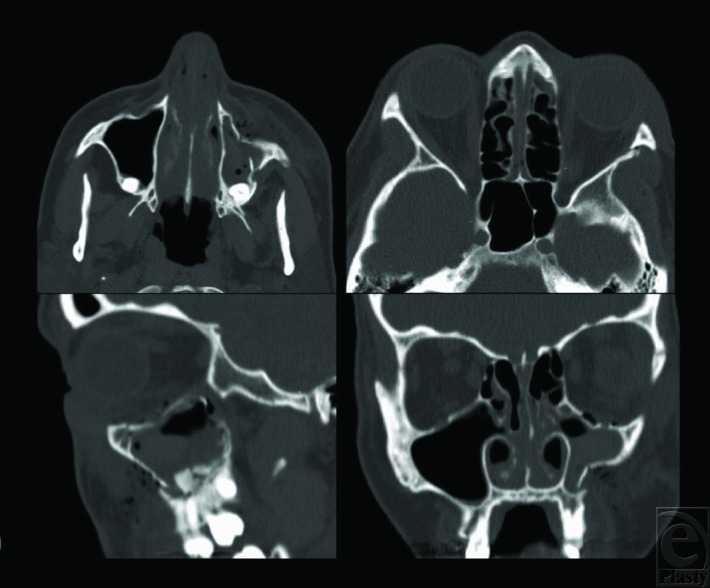
Maxillofacial computed tomographic scan, demonstrating a depressed zygomaticomaxillary complex fracture with loss of projection (top left), displacement at the sphenozygomatic suture (top right), zygomaticomaxillary buttress (bottom right), with minimal orbital floor displacement (bottom left).

Reduction should be performed for displaced fractures that result in trismus, contour asymmetry, or significant orbital floor disruption (Fig 1).^1^ The zygomaticomaxillary buttress can be accessed through a gingivobuccal sulcus incision. This incision is typically made 5 mm superior to the mucogingival junction through mucosa and then periosteum. The risks of this incision are damage to the parotid papilla, which is typically located adjacent to the first or second maxillary molar, and damage to the infraorbital nerve. The frontozygomatic suture can be accessed via an upper blepharoplasty incision or a lateral eyebrow incision. The upper blepharoplasty incision can easily be placed in the supratarsal fold and allows for an aesthetic scar, without the risk of brow alopecia that accompanies the lateral brow approach. If needed, the orbital floor can be accessed through a lower eyelid subciliary, subtarsal, or transconjunctival approach. The cutaneous approaches through the eyelid may offer better exposure, but carry the attendant risk of ectropion, which can be difficult to correct. A retroseptal, transconjunctival incision carries minimal risk of eyelid malposition and allows for exploration of the orbital floor and lateral wall (in conjunction with a lateral canthotomy).

Indications for orbital floor exploration and repair include extraocular muscle entrapment with limitation of upward gaze, comminution of the floor, enophthalmos more than 2 mm, defects posterior to the axis of the globe, or floor defects 5 to 10 mm on computed tomography.^6^ The orbital floor may be explored via lower eyelid approaches or endoscopically via a transantral approach.^4^^,^^6^ Confirmation of reduction of the sphenozygomatic suture can also be confirmed through this approach.

**Figure 2:**
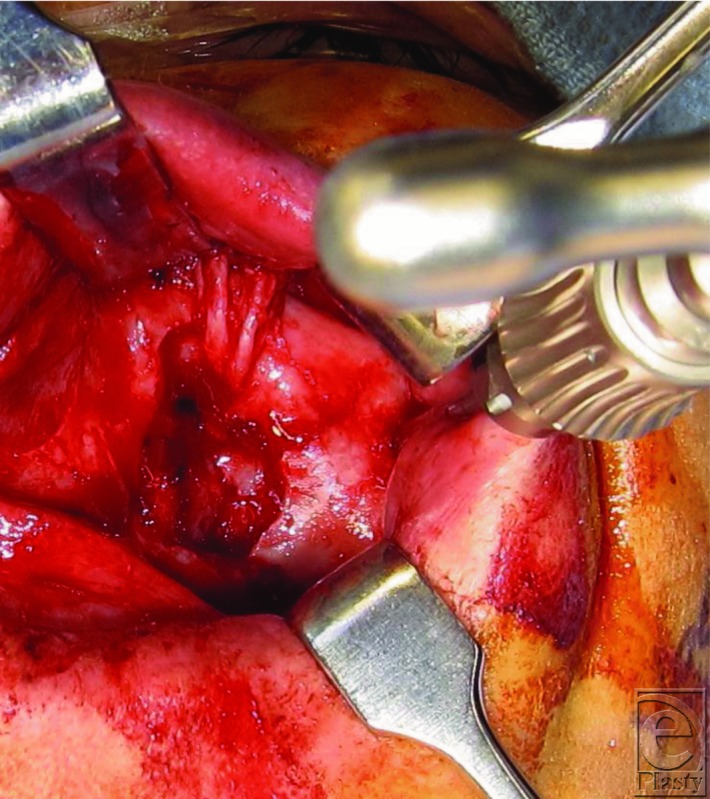
Clinical photograph demonstrating intraoral exposure of the zygoma, with a Carroll-Girard screw in place. The infraorbital nerve can be visualized medial to the screw.

**Figure 3:**
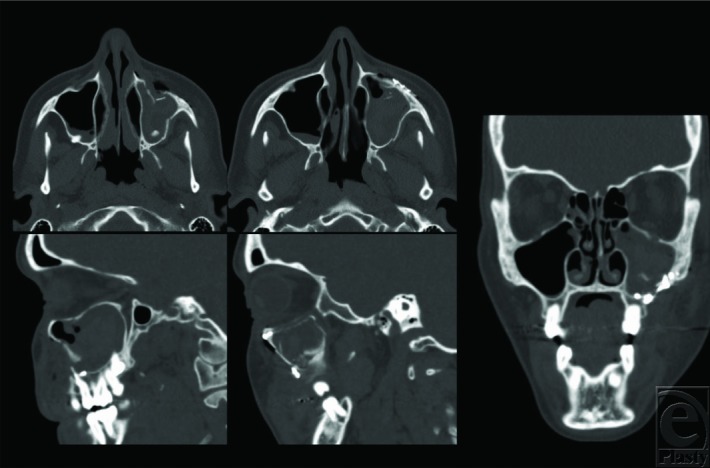
Maxillofacial computed tomographic scan, demonstrating reduction of the zygomaticomaxillary complex fracture. There is improved projection of the zygoma (top middle) compared with the preoperative state (top right). There is no significant increase in orbital volume or sizable floor defect (far right image and bottom images).

**Figure 4:**
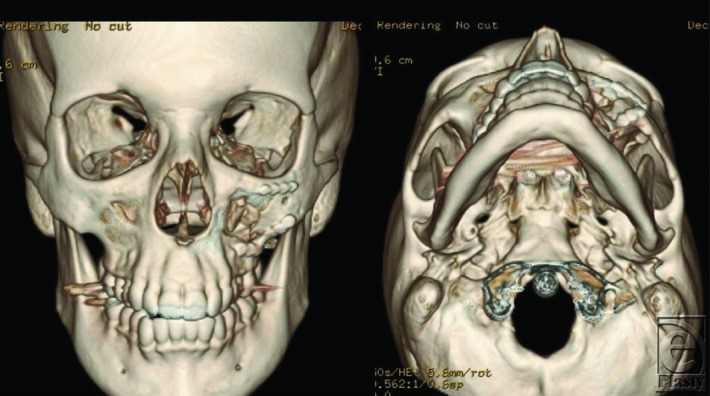
Three-dimensional reconstructions, demonstrating anatomic reduction of the zygomaticomaxillary complex fracture with hardware in place at the infraorbital rim and zygomaticomaxillary buttress.

This patient was diagnosed with a left zygomaticomaxillary complex fracture and underwent open reduction and internal fixation via a gingivobuccal sulcus approach. Reduction was achieved using a Carroll-Girard screw (Fig 2). Once reduction was established, fixation was accomplished using an L-shaped plate at the zygomaticomaxillary buttress and a curvilinear plate at the infraorbital rim after releasing and protecting the infraorbital nerve (arrow). Forced ductions were not suggestive of entrapment. Orbital floor exploration was not required. Postoperative imaging showed appropriate reduction of the ZMC (Figs 3 and 4).

## References

[1] Ellstrom CL, Evans GR (2013). Evidence-based medicine: zygoma fractures. Plast Reconstr Surg.

[2] Avery LL, Susarla SM, Novelline RA (2011). Multidetector and three-dimensional CT evaluation of the patient with maxillofacial injury. Radiol Clin North Am.

[3] Ellis E, Kittidumkerng W (1996). Analysis of treatment for isolated zygomaticomaxillary complex fractures. J Oral Maxillofac Surg.

[4] Olate S, Lima SM, Sawazaki R, Moreira RW, de Moraes M (2010). Surgical approaches and fixation patterns in zygomatic complex fractures. J Craniofac Surg.

[5] Kung DS, Kaban LB (1996). Supratarsal fold incision for approach to the superior lateral orbit. Oral Surg Oral Med Oral Pathol Oral Radiol Endod.

[6] Ellis E, Reddy L (2004). Status of the internal orbit after reduction of zygomaticomaxillary complex fractures. J Oral Maxillofac Surg.

